# Recognition of post-learning alteration of hippocampal ripples by convolutional neural network differs in the wild-type and AD mice

**DOI:** 10.1038/s41598-021-00598-8

**Published:** 2021-10-28

**Authors:** Sheng-Yi Hsu, Bartosz Jura, Mau-Hsiang Shih, Pierre Meyrand, Feng-Sheng Tsai, Tiaza Bem

**Affiliations:** 1 Department of Biomedical Imaging and Radiological Science, China Medical University, Taichung, 40402 Taiwan; 2 Research Center for Interneural Computing, China Medical University Hospital, Taichung, 40447 Taiwan; 3 Nalecz Institute of Biocybernetics and Biomedical Engineering, Polish Academy of Sciences, Ks. Trojdena 4, 02-109 Warsaw, Poland; 4 Neurocentre Magendie, INSERM U1215, University Bordeaux, Bordeaux, France; 5 Institute of Applied Psychology, Jagiellonian University, Cracow, Poland

**Keywords:** Learning and memory, Machine learning

## Abstract

Evidence indicates that sharp-wave ripples (SWRs) are primary network events supporting memory processes. However, some studies demonstrate that even after disruption of awake SWRs the animal can still learn spatial task or that SWRs may be not necessary to establish a cognitive map of the environment. Moreover, we have found recently that despite a deficit of sleep SWRs the APP/PS1 mice, a model of Alzheimer’s disease, show undisturbed spatial reference memory. Searching for a learning-related alteration of SWRs that could account for the efficiency of memory in these mice we use convolutional neural networks (CNN) to discriminate pre- and post-learning 256 ms samples of LFP signals, containing individual SWRs. We found that the fraction of samples that were correctly recognized by CNN in majority of discrimination sessions was equal to ~ 50% in the wild-type (WT) and only 14% in APP/PS1 mice. Moreover, removing signals generated in a close vicinity of SWRs significantly diminished the number of such highly recognizable samples in the WT but not in APP/PS1 group. These results indicate that in WT animals a large subset of SWRs and signals generated in their proximity may contain learning-related information whereas such information seem to be limited in the AD mice.

## Introduction

According to the current knowledge, hippocampal sharp wave-ripples (SWRs) are an important prerequisite for successful memory consolidation and decision making^1–6^. Indeed, experimental suppression of SWRs during slow-wave-sleep (SWS) impaired spatial memory formation^7,8^. SWRs involvement in memory processing is also indicated by observations that in many animal models of diseases with memory impairment a deficit of sleep and awake SWRs is observed^9–16^.

However, the animals can learn or perform a spatial task without the presence of awake SWRs^17^. Also, more recent studies using optogenetic blockage of SWRs suggest that they are dispensable for the formation of stable spatial representation in CA1^18^. In addition, we have recently shown that in APP/PS1 mice, a model of Alzheimer Disease (AD), SWRs are profoundly altered compared to their wild-type (WT) littermates despite the fact that APP/PS1 mice are able to consolidate spatial memory and correctly recognize the position of baited arms in 8 arm maze^19^. Namely, in APP/PS1 animals the occurrence rate of ripples has been diminished by 50% compared to the WT group. Moreover, the increase of the ripple rate that is typically observed after a learning session^19,20^ as well as a post-learning increase of the SWRs' intrinsic frequency^19^, was expressed only in the WT but not in the APP/PS1 animals. These drastic alterations of the APP/ PS1 ripple characteristics did not lead to an inability to encode and memorize the position of baited arms in this group.

Since hippocampal ripples mediate the retrieval of stored representation that can be used in different brain functions, like decision-making, planning, imagination, and consolidation of memory^4^, it cannot be excluded that within the reduced population of SWRs in APP/PS1 animals a specific type of ripples, which do play the role in the processing of recently encoded spatial information, was preserved. If so, at least some SWRs in APP/PS1 mice may have different characteristics before and after a learning session in the spatial memory task. Nevertheless, in this group, the comparison of SWRs' power, duration, and intrinsic frequency before and after learning did not reveal any differences^19^. We, therefore, choose to test whether deep learning algorithms are able to distinguish SWRs that were generated before from those that were generated after the learning session in this AD mice model.

## Results

**Figure 1:**
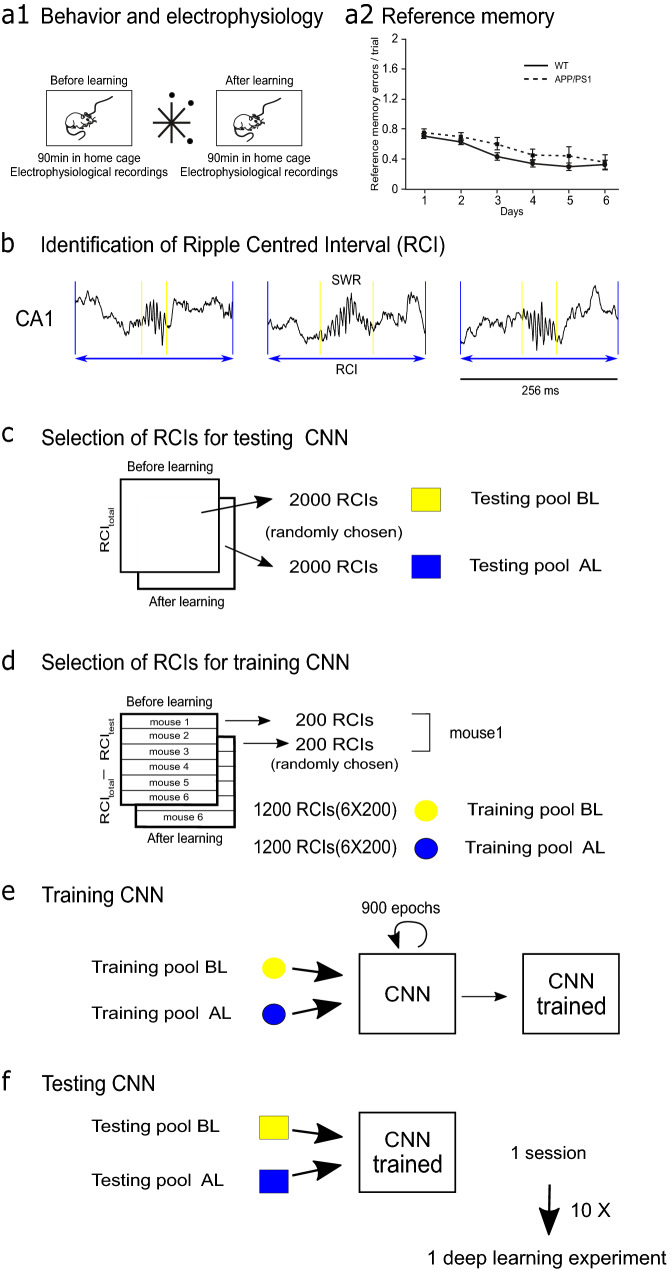
Schematic representation of behavioral and deep learning experiments. (**a1**) Electrophysiological recording was performed in the home cage during 90 min before and after visiting the maze, where the animal had to find the position of 3 baited arms. (**a2**) Acquisition of the reference memory was similar in WT and APP/PS1 groups. (**b–f**) 256 ms intervals of LFP signals recorded in CA1 during slow-wave sleep and centered at a single SWR (**b**) were randomly chosen to constitute the testing (**c**) and training data pools (**d**). Each of the 10 sessions of the deep learning experiment consisted of the training of CNN during 900 epochs on a new training pool (**e**) and the test on the same fixed test pool (**f**).

In earlier work^19^ we found a major deficit of SWRs occurring in APP/PS1 mice during slow-wave sleep, before and after learning session of a spatial memory task (Fig. 1a1) although the mice were able to consolidate spatial reference memory in a similar way as WT group (Fig. 1a2). These findings may question the role of SWRs in memory processing in this AD model. Interestingly, whereas SWRs generated before and after learning have different properties in the WT animals, no such differences have been so far found in the APP/PS1 group, further suggesting a lack of their involvement in memory processing. Searching for the putative learning-induced alteration of SWRs in the present study we test the capability of the CNN algorithms to recognize pre- and post-learning SWRs in both groups of animals. We used time windows of LFP signals recorded in CA1, containing SWRs (ripple-centered intervals, RCIs, see Fig. 1b).

As explained in the Material and Methods, 2000 RCIs were randomly chosen as a fixed testing pool of RCIs from the data recorded in each experimental condition (i.e. before or after learning) (Fig. 1c). From the remaining RCIs, a training pool was built using 200 randomly chosen RCIs from each animal in each experimental condition (Fig. 1d). After CNN's training, we tested its performance on the testing pool of RCIs (Fig. 1e,f). This procedure was repeated in 10 sessions, in each of which different training pools and the same testing pool were used.

In this experimental algorithm, an individual RCI can be correctly or incorrectly classified in each of 10 sessions. A percentage of correct classifications of a given RCI will therefore indicate a probability to be correctly recognized by CNN, which we define as accuracy rate (AR) attributed for a specific RCI. The average AR of RCIs for a given animal was calculated. Moreover, to better characterize the CNN's performance the percentage of highly recognizable RCIs (HR RCIs), that is RCIs correctly classified in at least 8 over 10 sessions, was calculated for each animal. The average AR and HR RCIs were obtained for each group of animals.

### Recognition of a post-learning alteration of RCIs is effective in WT but not APP/PS1 mice

**Figure 2:**
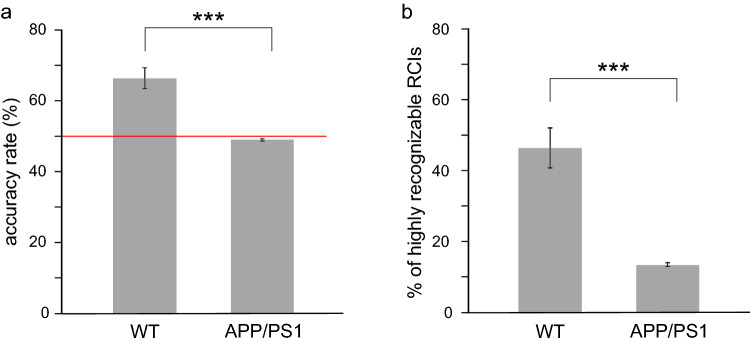
Recognition of a post-learning alteration of RCIs differs in the two groups. (**a**) Average accuracy rate was equal to the chance level in the APP/PS1 mice and significantly lower compared to the WT group (APP/PS1: 49%, WT: 66.29%, *p* = 0.0001, t = 6.0435, t-test, N = 6). (**b**) Whereas in the WT group almost half of tested RCIs was correctly recognized in at least 8 over 10 sessions, the percentage of such highly recognizable RCIs was nearly three times lower in APP/PS1 animals. (APP/PS1: 13.47%, WT: 46.39%, *p* = 0.0006, t = 4.9811, t-test, N = 6). All box plot represent mean values, vertical bars represent standard errors Abbreviations: *** *p* < 0.001, ** *p* < 0.01, * *p* < 0.05.

We first tested the capability of the CNN to classify RCIs in the control group of wild-type animals (N = 6). As illustrated in Fig. 2a, CNN was able to correctly identify a large population of RCIs in the testing pools. Indeed, the average AR in the WT group was equal to 66.29%, and 46.39% of RCIs were highly recognizable (Fig. 2b).

By contrast to a relatively good ability of the CNN to recognize RCIs in the WT group, the average AR for the APP/PS1 group was practically equal to the chance level (50%, see red line in Fig. 2a) and significantly lower than in WT animals (*p* = 0.0001, t = 6.0435, t-test, N = 6) (Fig. 2a). Moreover, the percentage of HR RCIs among all tested RCIs was equal only to 13.47% and was also significantly lower than in the WT group (*p* = 0.0006, t = 4.9811, t-test, N = 6) (Fig. 2b). The results of this deep learning experiment suggested that some learning-related information might be carried by SWRs in the WT group and therefore recognized by the CNN whereas in the APP/PS1 animals such information might be lacking.

### The difference in the pre- and post-learning SWRs' intrinsic frequency matters

**Figure 3:**
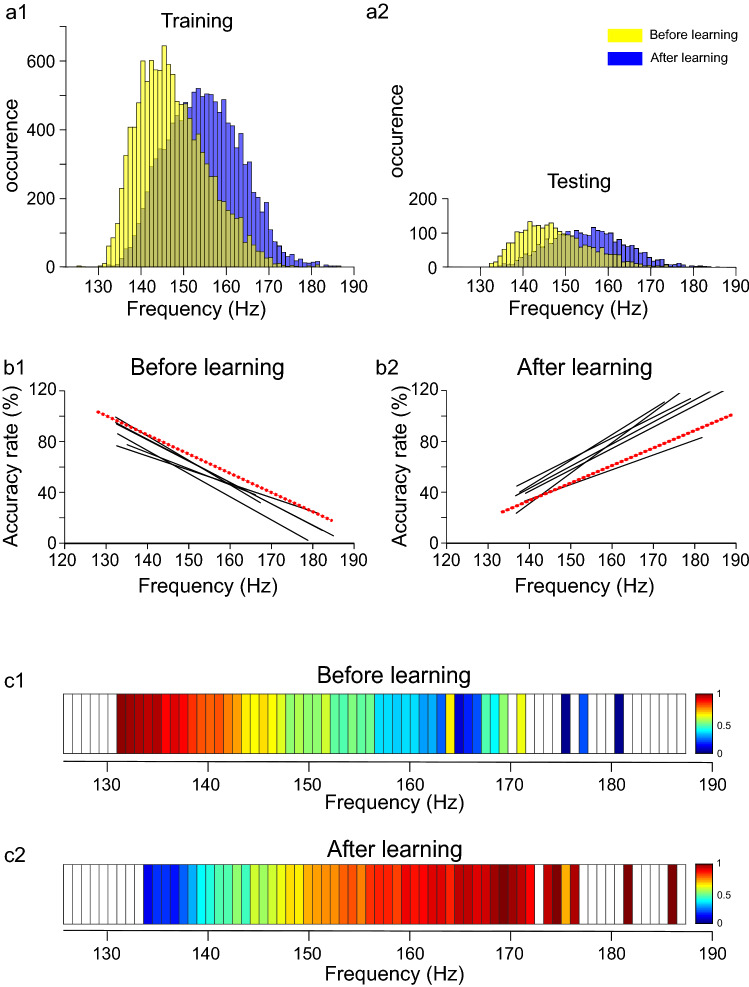
Accuracy rate of RCIs is correlated with the intrinsic frequency of SWRs in the WT mice. The post-learning increase of SWRs’ frequency was present both in the training (**a1**) and the testing pools of RCIs (**a2**). AR was correlated with SWRs’ frequency in individual animals (**b1–2**) as well as in the pooled set of RCIs (**c1–2**). Notice a positive correlation between SWR's frequency and AR of RCI belonging to "after" learning (see regressions lines in b2, color bars in **c2**) and a reverse relationship that was expressed "before" learning (see regressions lines in b1, color bars in **c1**). Regression lines indicated in black—correspond to individual animals, in red—to pooled RCIs. Color code is expressed in the percentage of AR (**c1–2**).

**Figure 4:**
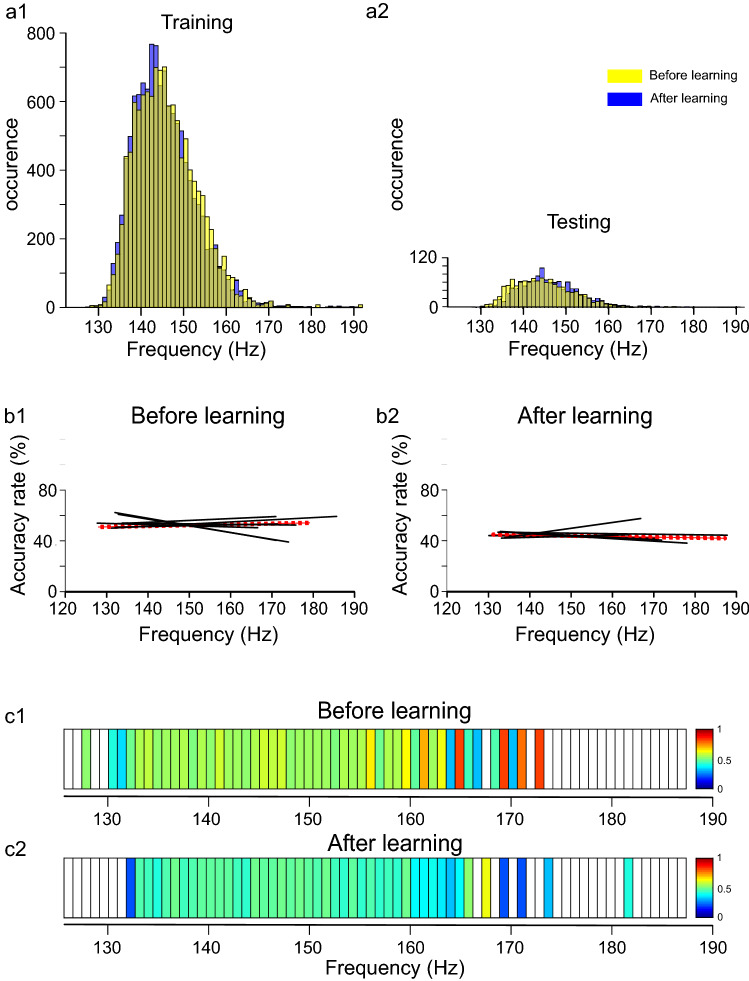
Accuracy rate of RCIs is not correlated with the intrinsic frequency of SWRs in the APP/PS1 mice. The SWRs’ frequency had similar distribution before and after learning both in the training (**a1**) and testing pools of RCIs (**a2**) and was correlated with RCI's accuracy rate neither in individual animals (**b1-2**) nor in the pooled set of RCIs (**c1-2**). Notice flat regression lines in b1-2 and the color code bars in c1-2 indicating an accuracy rate close to 50% independently on SWR's frequency. Other abbreviations as in Fig. 3.

As previously shown, SWR's intrinsic frequency increased significantly after a learning session in the WT but not in APP/PS1 mice^19^. It was, therefore, possible that the CNN was able to recognize RCIs in WT animals due to the difference in SWR's intrinsic frequency between "before" and "after" learning (cf. yellow and blue bars, respectively, Fig. 3a1–2) that was existing in the training and testing data pools. By contrast, CNN might fail to recognized RCIs in the APP/PS1 group due to an absence of such difference (cf. yellow and blue bars, Fig. 4a1–2). In order to test this hypothesis, we analyzed correlations between a given SWR's intrinsic frequency and AR of RCI containing this SWR.

We found a relatively high value of correlation coefficient R^2^ between SWR’s frequency and AR of RCIs belonging to “before learning” for each WT animal (R^2^ ranging from 0.1311 to 0.3685, N = 6) with regression lines showing negative slopes (slopes ranging from -1.28 to -2.61%/Hz, N = 6) (Fig. 3b1). This indicates that the lower the SWR's frequency the higher was the RCI's probability to be identified as belonging to the "before" data set. A reverse tendency was expressed in the set of RCIs recorded "after" learning (slopes ranging from 1.7 to 2.4%/Hz, R^2^ ranging from 0.1212 to 0.4117, N = 6): here the higher the frequency of SWR, the higher the probability of the RCI to be correctly recognized (Fig. 3b2). This is illustrated also for the pooled set of RCIs in the WT group (red regression lines, Fig. 3b1–2 and color-coded bars, Fig. 3c1–2), showing that correlation between SWR’s frequency and AR of RCI belonging to “before” (slope = -2.03%/Hz, R^2^ = 0.3268, see also Fig. 3c1) and “after” learning (slope = 1.84%/Hz, R^2^ = 0.3274, Fig. 3c2) was opposite. Altogether, these results indicate that in the WT group the CNN developed a successful strategy of classification of RCIs that was based on the intrinsic frequency of SWRs generated "before" and "after" learning.

Such a strategy could not be developed in the APP/PS1 mice since SWRs generated in this group have similar frequency distributions in the two experimental conditions (Fig. 4a1–2). Indeed, all regression lines illustrating the correlation between SWR's frequency and RCI's recognizability for APP/PS1 animals (N = 6) were characterized by a low value of the slope and correlation coefficient both in the set of data corresponding to "before" (slope ranging from − 0.53 to 0.2%/Hz; R^2^ ranging from 0.0001 to 0.0362) and “after” learning (slope ranging from − 0.21 to 0.09%/Hz; R^2^ ranging from 0.0002 to 0.0168) (Fig. 4b1,b2, respectively). Similar lack of correlation between SWR’s frequency and AR of RCI was found also for the pooled set of RCIs in the APP/PS1 group (“before”: slope = 0.06%/Hz, R^2^ = 0.0004, “after”: slope = 0.05%/Hz, R^2^ = 0.0003), as illustrated in Fig. 4b1–2 (red regression lines) and in Fig. 4c1–2.

**Figure 5:**
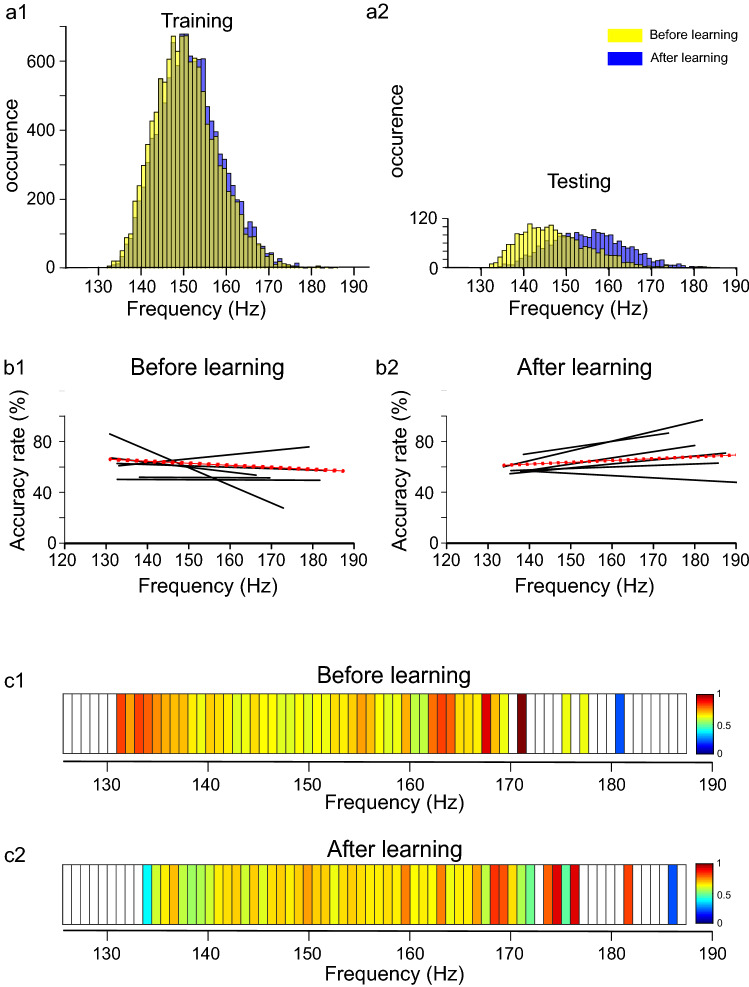
Equalizing distributions of the pre-and post-learning SWRs’ frequency in the training pool induces a new discrimination strategy in the WT group. When the SWRs’ frequency was chosen to have similar distribution before and after learning in the training (**a1**) but not in the testing pool of RCIs (**a2**) no correlation with RCI’s accuracy rate was expressed neither in individual animals (**b1–2**) nor in the pooled set of RCIs (**c1–2**), indicating not-frequency based classification of RCIs. Notice flattering of regression lines in b1-2 compared to Fig. 3 and color code bars in c1-2 indicating accuracy rate independent of SWR's frequency. Abbreviations as in Fig. 3.

In the next step, we asked whether the CNN is able to classify RCIs belonging to WT mice if the training pool (Fig. 5a1) is modified by requiring equal distribution of SWR's frequency corresponding to "before" and "after" learning (see the area of overlapping blue and yellow bars, Fig. 3a1), similarly as it was expressed in the APP/PS1 animals. (cf. Figs. 4a1, 5a1). We kept the testing pool unchanged, so SWR's frequency distributions "before" and "after" were different as in the previous deep learning experiment (Fig. 5a2). In this condition, we expected that the CNN's strategy could be based on some (unknown) features of RCIs, possibly related to the processing of the information encoded during learning, that are different from SWRs intrinsic frequency.

### Successful not frequency-based discrimination strategy in WT mice

As expected, the correlation between the AR of RCI and SWR’s frequency declined in this deep learning experiment. Indeed, all regression lines illustrating this relationship for individual animals in the WT group were characterized by low values of slopes and correlation coefficients R^2^ for RCIs belonging to “before” (slope ranging from − 1.39 to 0.33%/Hz, R^2^ ranging from 0.00001 to 0.1014) and “after” learning (slope ranging from − 0.17 to 0.76%/Hz, R^2^ ranging from 0.0015 to 0.0626) (Fig. 5b1–2). Also the pooled data set of RCIs did not express any correlation with SWR’s frequency in both experimental conditions (see red regression lines, Fig. 5b1–2), as indicated by low values of slopes (“before”: = − 0.16%/Hz; “after”: = 0.14%/Hz) and correlation coefficients (“before”: = 0.0023, “after”: = 0.002) as well as by the color-code representation in Fig. 5c1–2.

**Figure 6:**
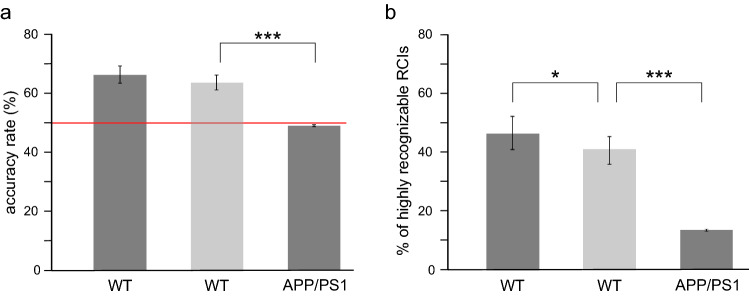
Successful not frequency-based classification of RCIs in the WT mice. **(a**,**b**) After equalizing the frequency distribution of the pre- and post-learning SWRs in the training pool the average AR (**a**) and HR RCIs (**b**) were still significantly higher in the WT than in APP/PS1 group (AR: *p* = 0.0004, t = 5.8035; HR RCIs: *p* = 0.0004, t = 5.8035, t-test with Bonferroni correction). However, in the WT group, the average value of HR RCIs was slightly lower compared to the experiment with full frequency (*p* = 0.0485, t = 3.1831, t-test with Bonferroni correction) (**b**). In the WT group, dark bars represent the mean value of AR and HR RCIs in the experiment with full frequency and light bars in the experiment with the equalized frequency of SWRs. Other abbreviations as in Fig. 2.

In this deep learning experiment, the CNN performed at the lower level than when trained on the data containing full information of SWRs' frequency, but still relatively well. Indeed, the value of the average AR as well as the percentage of HR RCIs in the WT group decreased only by ~ 5–10% compared to the previous experiment (AR: 66.29 + /− 0.03% (full frequency), 63.58 + /− 0.025% (equalized frequency), *p* = 0.0615, t = 2.9714, t-test with Bonferroni correction; HR RCIs: 46.39 + /− 5.56% (full frequency), 40.69 + /- 4.67% (equalized frequency), *p* = 0.0485, t = 3.1831, t-test with Bonferroni correction). Importantly, both the value of AR and percentage of HR RCIs were still significantly higher than the performance of the CNN in the APP/PS1 group (AR: *p* = 0.0004, t = 5.8035; HR RCIs: *p* = 0.0004, t = 5.8035, t-test with Bonferroni correction) (Fig. 6).

### The distribution of recognized RCIs differs in the two groups of animals

**Figure 7:**
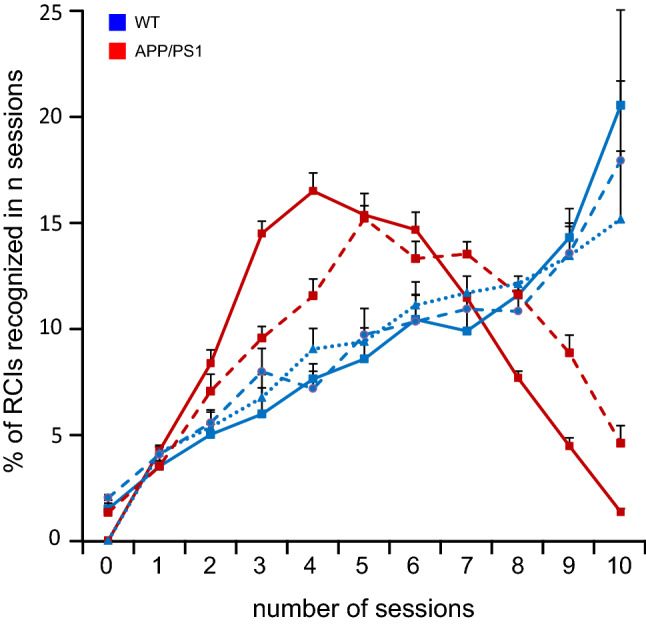
Distribution of recognized RCIs. The percentage of RCIs correctly recognized in a given number of sessions was plotted from 0 to 10 sessions in the WT (blue lines) and APP/PS1 group (red lines). Distributions in 3 experiments are indicated as follows: full frequency distribution of SWRs and full RCI content—solid lines, equalized distribution of SWRs' frequency and full RCI content—dotted line, a full frequency distribution of SWRs and RCI without head and tail—dashed lines. Notice the remarkable difference in the distribution shape in the two groups: Whereas in the WT mice, the number of correctly recognized RCIs increases monotonically with the number of sessions considered (see solid, dotted, and dashed blue lines), in the APP/PS1 mice the distribution has a gaussian-like shape with a pronounced tendency for symmetry between the number of sessions with correct and in-correct recognition.

So far, only highly recognizable RCI, correctly classified in at least 8 sessions, were analyzed. To illustrate completely the CNN’s performance in the three deep learning experiments described above we calculated the percentage of RCIs correctly classified in any number of sessions (from 0-not recognized in any session, to 10—recognized in all sessions) (Fig. 7). As illustrated, the CNN’s performance in the two deep learning experiments on the WT group, with full and equalized SWR’s frequency distribution in the training pool, was similar (see, blue solid and dotted lines, respectively, Fig. 7). Indeed, in both experiments the percentage of RCIs correctly classified increased monotonically with the number of sessions considered (see from n = 0–10, Fig. 7). By contrast, the distribution characterizing performance of the CNN in the APP/PS1 group had a different shape, suggesting a random process of RCIs classification. Indeed, the distribution was fairly symmetrical, with most of RCIs being classified correctly 4–6 times (see the maximal value of the red solid curve, Fig. 7), which implicates the same number of incorrect classifications.

Altogether these results show that the CNN trained on RCIs containing information about the difference of the pre- and post-learning SWRs' intrinsic frequency developed a successful classification strategy that was based on this difference (or on some RCIs' features which occurrence was correlated with SWRs' frequency). While trained on the data set not containing information about the difference of the SWRs' intrinsic frequency in the two experimental conditions, the CNN developed a different strategy of RCIs' classification, also relatively successful, that was based on some unknown features of the RCIs, that were different before and after learning and independent of SWRs' frequency. Contrary to a good and flexible CNN's ability of correct discrimination of pre- and post-learning RCIs in the WT group the CNN failed to classify RCIs belonging to the APP/PS1 animals, which suggested a lack of learning-dependent information in this pool of data.

### Elimination of the head and tail of RCI has an opposite effect in the two groups of mice

**Figure 8:**
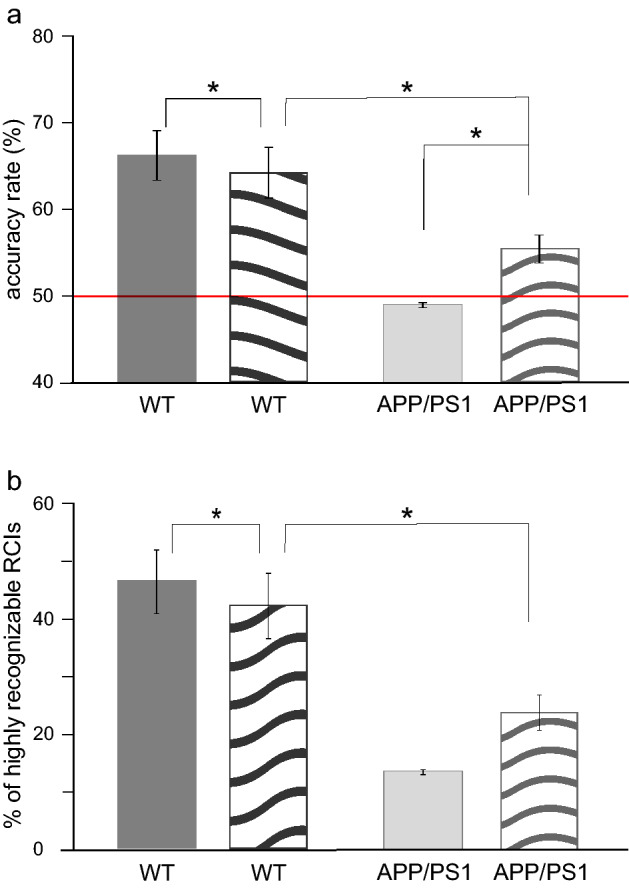
Elimination of the head and tail of RCI has an opposite effect on the CNN’s performance in the WT and APP/PS1 group. (**a**,**b**) In the WT group reduction of the analyzed signals to SWRs alone resulted in decreasing of the average AR (*p* = 0.027, t = 4.1487, t-test with Bonferroni correction) (**a**) and HR RCIs (*p* = 0.031, t = 3.9947, t-test with Bonferroni correction) (**b**) compared to the experiment with the full content of RCI exposed to analysis. A reverse effect was expressed in the APP/PS1 group as illustrated by the increase of the mean value of AR (*p* = 0.011, t = 5.1624, t-test with Bonferroni correction) (a). Notice however a lack of a significant change of HR RCIs in this group (*p* = 0.054, t = 3.4631, t-test with Bonferroni correction). Importantly, the mean value of HR RCIs was still significantly higher in the WT compared to APP/PS1 animals (*p* = 0.049, t = 2.869, t-test with Bonferroni correction) Dark bars represent average values of AR and HR RCIs in the experiment with the full content of RCIs, patterned bars correspond to the experiment with the elimination of the head and tail of RCIs. Other abbreviations as in Fig. 2.

As demonstrated by the recent study, some memory processing may occur in the close vicinity of SWRs (see 4). It was therefore interesting to test whether removing the information carried in the neighborhood of SWRs may reduce the ability of the CNN to classify RCIs. To this aim, in each RCI, we replaced signals recorded prior and posterior to SWR by zeros (no information), both in the training and testing pools of the WT and the APP/PS1 group. As illustrated in Fig. 8, in WT animals eliminating LFP signals at the close vicinity of SWRs resulted in a diminution of the CNN's performance and significantly reduced the mean values of AR (full RCI: 66.29%, only SWR: 64.24%, *p* = 0.027, t = 4.1487, t-test with Bonferroni correction) (Fig. 8a) and the percentage of HR RCIs (full RCI: 46.39%, only SWR: 42,29%, *p* = 0.031, t = 3.9947, t-test with Bonferroni correction) (Fig. 8b) indicating that removed signals contained pertinent information for the CNN’s classification process. Surprisingly, the same procedure resulted in a significant improvement of the CNN performance in the APP/PS1 group, as illustrated by the increased mean value of AR (full RCI: 49%, only SWR: 55,53%, *p* = 0.011, t = 5.1624, t-test with Bonferroni correction) (Fig. 8a) although the percentage of HR RCIs remained still not significantly changed compared to recognition of full continent of RCIs (full RCI: 13.47%, only SWR: 23,81%, *p* = 0.054, t = 3.4631, t-test with Bonferroni correction) (Fig. 8b). These results indicate that in the APP/PS1 group signals occurring prior and posterior to SWRs contained no information that could be useful in the discrimination of pre- and post-learning RCIs and eliminating them enable the CNN to improve a classification strategy. Importantly, while the CNN was analyzing only the content of SWRs, although the resulting AR was similar to the level expressed in APP/PS1 mice (*p* = 0.076, t = 2.607, t-test with Bonferroni correction) the percentage of HR RCIs was still significantly higher in the WT compared to APP/PS1 animals (*p* = 0.049, t = 2.869, t-test with Bonferroni correction) (Fig. 8). Moreover, the distribution of correctly classified RCIs did not change considerably and was still gaussian-like shaped in the APP/PS1 group and monotonically increasing with the number of sessions in the WT group (see dashed blue and dashed red lines, respectively, Fig. 7).

## Discussion

In this study we compare the performance of CNN algorithms to discriminate pre- and post-learning RCIs in the two groups of related animals, namely, the APP/PS1 mice which are a model of Alzheimer disease (AD) and their littermate kin (wild-type, WT).

We found that whereas CNN efficiently recognized RCIs as belonging to pre- or post-learning sessions in the WT group, it failed to do so in the APP/PS1 mice. Indeed, whereas the mean AR of SWRs belonging to the WT group was equal to 66%, in the APP/PS1 animals it was at the chance level (50%), suggesting that CNN's classification process was randomized in this group. This was further supported by a difference between the two groups in the distribution of RCIs correctly recognized in a given number of sessions. Whereas in APP/PS1 animals this distribution had a gaussian-like shape, indicating a random process of RCIs' classification, in the WT group the number of recognized RCIs increased with their recognizability (i.e. with the number of sessions in which they were correctly recognized) (Fig. 7). Consequently, in the WT group, the number of highly recognizable RCIs was equal to almost 50% of tested RCIs whereas in APP/PS1 group this amount was less than 15%. These findings indicated that the CNN developed a successful strategy to discriminate pre- and post-learning RCIs in the WT group. By contrast, it was unable to find learning-related features of RCIs in the APP/PS1 mice, which were nevertheless successful in memorizing a spatial task^19^.

However, since the intrinsic frequency of pre- and post-learning SWRs differed in the WT but not APP/PS1 mice we performed another deep learning experiment, in which we tested the CNN’s discrimination capability after being trained on RCIs with equalized pre- and post-learning frequency distributions of SWRs. It is noteworthy that the CNN’s performance in this experiment was still considerably better than in the APP/PS1 group (Fig. 6). These results demonstrated that the CNN was able to develop two different classification strategies in the WT group, based on RCIs’ features which occurrence was correlated, or not correlated, with the SWRs’ frequency, depending on the training data pool, but failed to find a successful algorithm allowing to discriminate pre-and post- learning RCIs in the APP/PS1 mice. This reinforces the idea that in the APP/PS1 mice, although the hippocampal neural networks are still able to generate SWRs, these latter seem to be incapable to be altered by learning.

Whereas a clear difference in the CNN's ability to discriminate pre-and post-learning RCIs was found between the two groups of animals, reducing the information content of RCIs by removing pre- and post- SWR signals had a puzzling effect on the CNN's performance. Indeed, in the first two deep learning experiments, the CNN analyzed a full content of RCI of the duration of 256 ms, in which SWR occupied only approximately 25%. This proportion was chosen in order to test a putative contribution of signals generated in the close vicinity of SWR on the CNN's performance. As reported recently, disrupting neural activity following SWRs by light stimulus disrupted hippocampal-dependent memory, suggesting that these signals contribute to spatial learning^21^. Moreover, a cortical-hippocampal-cortical loop of information transmission, possibly involved in memory consolidation, was recently postulated around the time of sleep SWRs, in which patterns of cortical activity was found to be predictable for subsequent SWRs activity, that in turn initiated activation of multiple cortical areas^22,23^. Obviously, cortical signals were not involved in the CNN classification process in our experiments. Interestingly, however, in the WT group, we did found a negative effect of the elimination of CA1 signals proceeding or following SWRs on the CNN's performance, indicating their alteration by a process of learning. By contrast, in APP/PS1 animals such elimination resulted in a slight but significant improvement of the CNN's performance (Fig. 8a). This indicates that the pre- and post-SWRs signals in the APP/PS1 mice were not useful in the CNN classification process which in turn could be upgraded by reducing the amount of information to be processed. Interestingly, removing SWRs from RCIs with the remaining content conserved resulted in the complete incapability of CNN to perform a correct classification in the WT group (AR at the chance level, data not shown). This suggests that in WT, but not APP/PS1 animals, a synergy between SWR per se and surrounding signals occurs and can be recognized by the CNN whereas in APP/PS1 mice only a very content of SWRs preserved a relevant information that can be used in the classification task.

In the WT group highly recognizable SWRs, that is SWRs correctly classified in at least 8 of 10 testing sessions and possibly altered by learning, constituted not more than 50% of all the population. There are some factors that may influence this proportion. First, apparently not all SWRs generated after a given learning session must carry information about the session. Indeed, only 10 to maximally 40% of SWRs were found to be involved in a replay of activity patterns representing past experience^24–26^. Second, the data pools corresponding to "before" and "after" learning were collected from all 6 days of learning in the 8-arm maze. In days 2–6, "before" learning, the animals were placed in experimental conditions similar to previously experienced, with the same view of the experimental room and the maze. This might activate previously formed memories of a visit in the maze and mark the content of some RCIs, which thus could become somehow similar to those generated "after" learning.

Finally, the most striking results obtained in our study is the amount of RCIs that have been correctly recognized using just LFP signals as belonging to pre- or post-learning sessions in the WT group. More than 40% of RCIs were found to be highly recognizable even if the information concerning SWRs' frequency available during CNN's training was not complete and might have been misleading and almost 50%—when the information was fully available. Whereas it is hippocampal spiking during SWRs which represents previous experience, no spiking patterns could have been recognized by the CNN in LFP signals recorded in this study and possibly used as a key signature of post-learning RCIs. Therefore there must exist some features of RCIs, different from SWR's intrinsic frequency and spiking patterns, that are profoundly altered after learning sessions in approximately half of RCIs. What these features are and how they are related to the memory processing remains to be revealed in the future study.

## Material and methods

### Animals and surgery

The electrophysiological data analyzed in this study were taken from our previous work^19^. The mice used were double transgenic APP/PS1 mice—a mouse model of Alzheimer Disease (AD) combining cognitive and amyloid pathologies starting at 4 months old as previously reported ^27^ and their wild-type (WT) littermates. The APP/PS1 model resulting from the crossing of 2 lines of commercial simple transgenic mice: APPswe, Tg2576, and PS1dE9 has received ethical authorization # 3804 and 21377 from CEEA50, Bordeaux. The genotypes of animals were controlled by a polymerase chain reaction of tail biopsy. Data were collected from 6 APP/PS1 and 6 WT females (8–9 months old). The animals were held in the animal facility as described in 19. Although electrodes were implanted in different cortices as well as in dorsal hippocamp in the present study only electrophysiological activity recorded in the CA1 area was analyzed. For the implantation stereotaxic surgery under deep isoflurane anesthesia was used.

Electrodes, consisting of insulated tungsten wire (diameter 35 μm, California Fine Wires), were implanted using stereotaxic coordinates into the CA1 region of left and right hippocampus (AP: + 2.0 mm, L: −/ + 1.5 mm (left or right hemisphere), VD: − 1.05 mm ), reference and ground electrodes were implanted into the cerebellum. The electromyogram (EMG) electrode was inserted into the neck muscles. The animals were housed individually during 3–4 weeks after surgery before the beginning of recordings and behavioral sessions (see 19 for more details). Experimental procedures complied with official European Guidelines for the care and use of laboratory animals (directive 2010/63/UE) and were approved by the ethical committee of the University of Bordeaux (protocol A50120159 and A16323). Before starting spatial memory tests mice were gradually food restricted to maintain their body weight at 85% of their ad libitum body weight. During the course of experiments, their access to water remained free. All procedures took place during the light cycle.

### Behavioral experiments

Spatial memory of the animals was tested in an eight-arm radial maze, as described previously^19^. Briefly, to provide a spatial hippocampal-dependent learning task various distal cues were positioned on the walls of the experimental room. Mice were familiarized with the radial maze and its environment during two days of habituation. As illustrated in Fig. 1a1, at the start of each daily experimental procedure, food-restricted mice stayed in the home cage with the connector plugged into the recording system for 90 min. Then, they were disconnected and placed into the maze where 3 arms were baited with food rewards. The trial was ended when all the rewards were eaten. Each animal performed six trials per daily session. After performing the trials animals were placed back to their home cage where they stayed 90 min connected to the recording system. The same procedure was used for 6 consecutive days.

### Data acquisition and processing

During the recording session, the mouse head connector was linked to amplifiers by a soft cable allowing free motions of the animal. Behavior was tracked with a video camera. Neurophysiological and EMG signals were acquired at 40 kHz on a 128-channel Plexon system and stored on a PC for offline analysis. Data were down-sampled to 2000 Hz using Matlab's 'decimate' procedure. Identification of REM and slow-wave sleep (SWS) was performed by visual inspection using EMG, spectrograms, delta/theta ratios as well as video-recording, as previously described^19^. Filtering of the signals was performed using Chebyshev Type II filter (order 4). Episodes of SWRs were detected in signals filtered in the 100–250 Hz frequency band. Envelopes of the narrow-band filtered signal, calculated using the Hilbert transform were z-scored. SWRs bouts were identified as epochs in which the envelope exceeded 2 SDs of the signal and reached 5 SDs, with the time points of the 2 SDs-crossing taken as the onset and offset of the SWR. SWRs spaced less than 20 ms apart were merged and longer than 100 ms were discarded. SWRs intrinsic frequency was calculated using a Hilbert transform of the signal.

### Deep learning experiments

To test the capability of the deep learning algorithms to recognize pre- and post-learning SWRs we used selected time windows of LFP signals recorded in CA1 during SWS, while the animal stayed in the home cage, before and after the session of a spatial memory task. As illustrated in Fig. 1b each selected interval of the duration of 256 ms was centered at a single SWR. These ripple-centered intervals (RCIs) were not overlapping and separated by at least 10 ms from neighboring SWRs. RCIs pool from the data recorded in a given experimental condition (before or after learning) in all animals of a given group (WT or APP/PS1) was used to generate testing and training pools of RCIs (Fig. 1c,d). First, 2000 RCIs were randomly chosen as the testing pool for each experimental condition. From remaining RCIs a specific training data set was extracted using 200 randomly chosen RCIs from each animal in a given experimental conditions (1200 for a group and condition). After 900 epochs of training CNN on this training pool (Fig. 1e), its performance was tested on the testing pool (Fig. 1f). Such a procedure is defined as one deep learning session. Since the training of CNN in one session involved certain random processes in initializing the weights of CNN, collecting RCIs in batches, and updating gradient descents, for one deep learning experiment we performed 10 sessions as random cross-validation, each consisting of the CNN’s training with a new set of randomly chosen RCIs and a test on the same fixed data set (Fig. 1f).

### CNN

The general approach of a classification task in machine learning is to build a model *M*_*w*_ with a weight vector *w* that gives a prediction vector *y* while receiving an input vector *x*, that is, $y = M_{w} \left( x \right)$. In our case, the vector *x* stands for the input RCI, and the vector *y* is a probability distribution vector indicating the classification of *x* belonging to “after” learning (AL) or “before” learning (BL). A loss function $L\left( {y,\tilde{y}} \right)$ is chosen to calculate the error between the prediction *y* and the ground truth $\tilde{y}$ corresponding to the class to which *x* actually belongs. To minimize the loss, one may search a suitable weight vector *w* by applying the gradient descent (GD) method to the loss:

$$ w\left( {t + 1} \right) = w\left( t \right) - \alpha \nabla_{w} L\left( {y\left( t \right),\tilde{y}\left( t \right)} \right), $$

where *t* is the iteration index, $\nabla_{w}$ is the gradient operator with respect to the variable *w*, and $\alpha$ is the learning rate that controls the convergence speed for the iteration. In our work, we chose the CNN algorithm to be the model classifying pre- and post-learning SWRs. Indeed, the CNN performed much better, with a greater than 10% average accuracy rate, than other deep learning algorithms, such as fully connected networks^28,29^ or recurrent neural networks(^30–32^).

**Figure 9:**
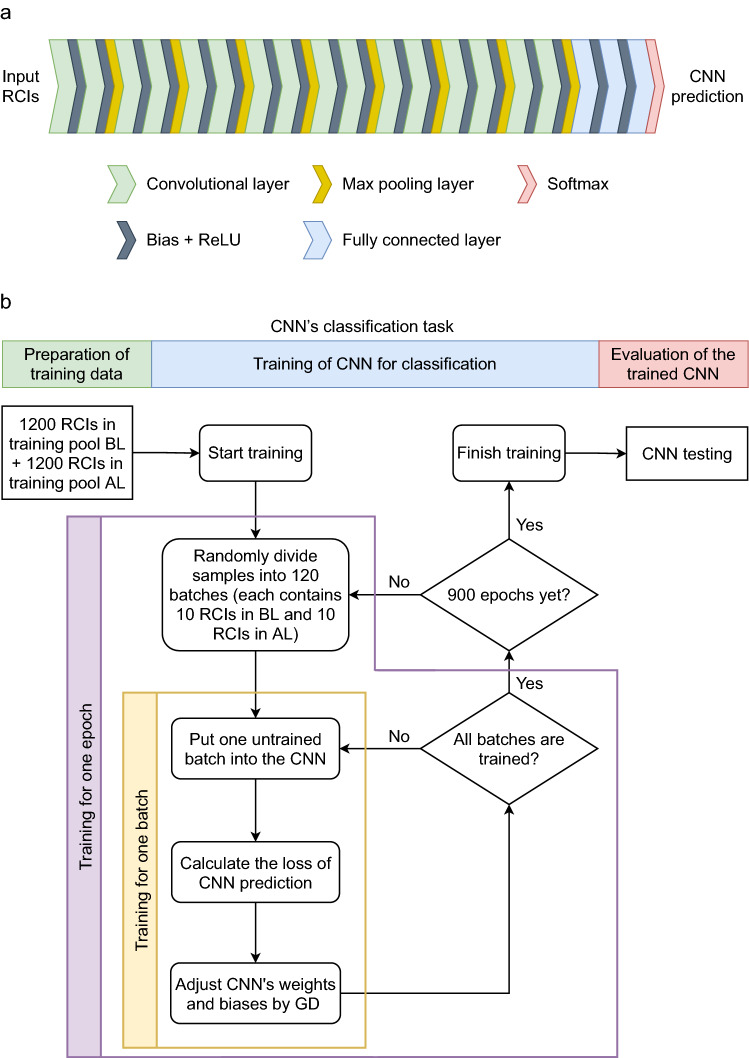
CNN. (**a**) The CNN constructed of convolutional layers, max pooling layers, and fully connected layers returns a prediction vector indicating the classification of the input RCI belonging to “after” learning (AL) or “before” learning (BL). (**b**) The CNN’s classification task is composed of the preparation of training data, the training of CNN for classification, and the evaluation of the trained CNN. Training CNN for a batch of RICs requires the calculation of GD of the loss of predicted results, which in turn adjusts CNN's weights and biases to improve the performance of CNN. After one epoch of training throughout all batches of RCIs, we redistribute RCIs randomly into batches and initiate the next generation of batch training. We stop training CNN after 900 epochs and then test its classification accuracy.

One could simply construct a CNN model as layers of modules/functions taking the output of the previous layer as its input, i.e., a composition of functions^33^. Accordingly, the CNN we used consists of three kinds of modules, namely, convolutional layers, pooling operations, and fully connected layers (Fig. 9a). Each module is a function taking vectors as its input and output. A convolutional layer acts as following

$$ y_{k} = \sum\nolimits_{m} {x_{k + m} w_{m} } , $$

where $x_{i}$, $y_{i}$, and $w_{i}$ correspond to the components of the input, output, and weight vectors of the convolutional layer, respectively. The pooling operation we adopt is max pooling (Fig. 9a) which acts as.

$$ y_{k} = \max \left\{ {x_{2k - 1} ,x_{2k} } \right\}, $$

where $x_{i}$ and $y_{i}$ also stand for the components of the input and output vectors, respectively. The fully connected layer is simply outputting the result of the input vector multiplied by the weight matrix: $y_{k} = \sum\nolimits_{m} {x_{k,m} w_{m} }$. A bias term is usually added to the output of convolutional layers and fully connected layers, and then followed by a rectified linear unit (ReLU) (Fig. 9a) defined by

$$ {\text{ReLU}}\left( {y_{k} + b_{k} } \right) = \max \left\{ {0,y_{k} + b_{k} } \right\}, $$

where $b_{k}$ stands for the bias and $y_{k}$ stands for the component of the output vector. These terms make the whole CNN model nonlinear, which is essential to achieve a general classification task.

In this work, the CNN was designed as 16 convolutional layers in total, with 8 max pooling operations performed between convolutions, 2 fully connected layers equipped with rectifier functions, and 1 fully connected layer equipped with the softmax function (Fig. 9a). The softmax function converts the output vector into a probability distribution vector whose *k*th component is defined by

$$ {\text{softmax}}\left( {y_{k} } \right) = \frac{{e^{{y_{k} }} }}{{\sum\nolimits_{i} {e^{{y_{i} }} } }}, $$

within which the component of the maximal softmax is referred to as the predicted class of the input x. The batch size is equal to 40 and the loss function is defined accordingly by

$$ - \frac{1}{40}\sum\limits_{m = 1}^{40} {\log \left\langle {y^{m} ,\tilde{y}^{m} } \right\rangle } , $$

where $\left\langle {y^{m} ,\tilde{y}^{m} } \right\rangle$ denots the usual inner product of the ground truth vector $\tilde{y}^{m}$ and the probability distribution vector $y^{m}$ generated from the outcomes of the CNN. The loss function quantifies the performance. The loss for the best recognition of RCIs is equal to 0. The loss is descended gradually by taking gradient updates on the weights and biases of CNN, as explained by the flow chart of training CNN (Fig. 9b).

The study is reported in accordance with ARRIVE guidelines.
